# Barriers and enablers in the implementation of a quality improvement program for acute coronary syndromes in hospitals: a qualitative analysis using the consolidated framework for implementation research

**DOI:** 10.1186/s13012-022-01207-6

**Published:** 2022-06-01

**Authors:** Shuduo Zhou, Junxiong Ma, Xuejie Dong, Na Li, Yuqi Duan, Zongbin Wang, Liqun Gao, Lu Han, Shu Tu, Zhisheng Liang, Fangjing Liu, Kenneth A. LaBresh, Sidney C. Smith, Yinzi Jin, Zhi-Jie Zheng

**Affiliations:** 1 Department of Global Health, School of Public Health, Peking University, 38 Xue Yuan Road, Haidian District, Beijing, 100191 China; 2 Institute for Global Health and Development, Peking University, 38 Xue Yuan Road, Haidian District, Beijing, 100191 China; 3 RTI International, Waltham, MA USA; 4 Division of Cardiovascular Medicine, School of Medicine, University of North Carolina at Chapel Hill, Chapel Hill, NC USA

**Keywords:** ACS, Quality improvement, Barriers, Enablers, CFIR, China, Qualitative study

## Abstract

**Background:**

Ischemic heart disease causes a high disease burden globally and numerous challenges in treatment, particularly in developing countries such as China. The National Chest Pain Centers Program (NCPCP) was launched in China as the first nationwide, hospital-based, comprehensive, continuous quality improvement (QI) program to improve early diagnosis and standardized treatment of acute coronary syndromes (ACS) and improve patients’ clinical outcomes. With implementation and scaling up of the NCPCP, we investigated barriers and enablers in the NCPCP implementation process and provided examples and ideas for overcoming such barriers.

**Methods:**

We conducted a nationally representative survey in six cities in China. A total of 165 key informant interviewees, including directors and coordinators of chest pain centers (CPCs) in 90 hospitals, participated in semi-structured interviews. The interviews were transcribed verbatim, translated into English, and analyzed in NVivo 12.0. We used the Consolidated Framework for Implementation Research (CFIR) to guide the codes and themes.

**Results:**

Barriers to NCPCP implementation mainly arose from nine CFIR constructs. Barriers included the complexity of the intervention (complexity), low flexibility of requirements (adaptability), a lack of recognition of chest pain in patients with ACS (patient needs and resources), relatively low government support (external policies and incentives), staff mobility in the emergency department and other related departments (structural characteristics), resistance from related departments (networks and communications), overwhelming tasks for CPC coordinators (compatibility), lack of available resources for regular CPC operations (available resources), and fidelity to and sustainability of intervention implementation (executing). Enablers of intervention implementation were inner motivation for change (intervention sources), evidence strength and quality of intervention, relatively low cost (cost), individual knowledge and beliefs regarding the intervention, pressure from other hospitals (peer pressure), incentives and rewards of the intervention, and involvement of hospital leaders (leadership engagement, engaging).

**Conclusion:**

Simplifying the intervention to adapt routine tasks for medical staff and optimizing operational mechanisms between the prehospital emergency system and in-hospital treatment system with government support, as well as enhancing emergency awareness among patients with chest pain are critically important to NCPCP implementation. Clarifying and addressing these barriers is key to designing a sustainable QI program for acute cardiovascular diseases in China and similar contexts across developing countries worldwide.

**Trial registration:**

This study was registered in the Chinese Clinical Trial Registry (ChiCTR 2100043319), registered 10 February 2021.

**Supplementary Information:**

The online version contains supplementary material available at 10.1186/s13012-022-01207-6.

Contributions to the literature

- To our knowledge, this qualitative study was the first process evaluation of an ongoing national quality improvement program for acute coronary syndromes.
- Barriers affect the ability of the National Chest Pain Centers Program to translate into effective patient outcomes. We aimed to identify barriers and enablers in the implementation process and provide ideas for overcoming these barriers.
- The findings of this study will generate actionable information to guide the design of impactful and sustainable quality improvement initiatives for acute coronary syndromes in China and other developing countries.

## Introduction

Ischemic heart disease (IHD) is the leading cause of death worldwide [1]. IHD has been increasing rapidly over the past 10 years [2]. With the increasing prevalence of IHD among younger populations [3, 4], approximately 40% of IHD-related deaths are premature [5], causing profound social and economic consequences for developed and developing countries [6, 7]. Most IHD deaths are from acute coronary syndromes (ACS), particularly in developing countries such as China [8]. Previous studies have identified numerous challenges in ACS treatment, including systematic delays and poor and unequal quality across different regions [9–13]. Time is critical for patients, and early diagnosis and treatment following clinical pathways are crucial to improving ACS care quality [14]. Many quality improvement programs use single or multiple strategies, including clinical pathway training, auditing care delivery, evaluating performance, and providing feedback to fill gaps in ACS care quality [15–17]. However, the results of these approaches remain suboptimal, with most interventions improving process quality indicators, such as drug use, but not outcome quality, especially in-hospital mortality and major adverse cardiovascular events [18, 19].

Many interventions fail to translate into effective patient care outcomes [20]. More than 60% of projects aimed at improvement have not resulted in organizational change owing to multilevel barriers from individuals, groups, organizations, and healthcare systems [21, 22]. For health services researchers, implementation science is becoming increasingly important. The role of implementation science is to provide the mechanism by which interventions affect outcomes via formative evaluations [23]. To the best of our knowledge, most evaluation studies of quality improvement programs have focused on endpoint health outcomes [24–26], with little research into influencing factors within the implementation process, particularly in China, which has one of the highest IHD disease burden in the world and various healthcare reforms aimed at improving care quality [27].

**Fig. 1:**
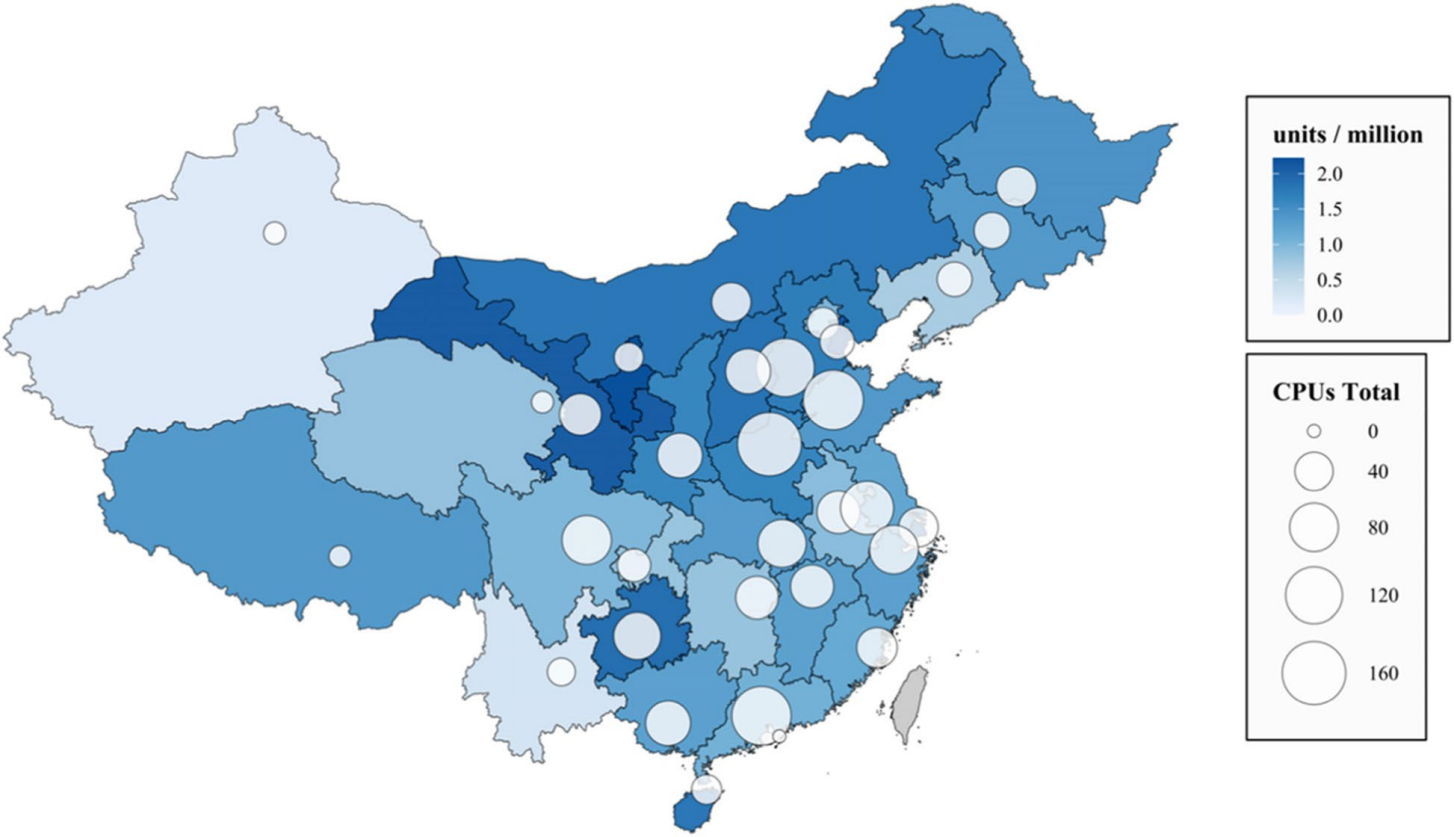
Distribution of CPCs in China. CPC, chest pain center

Accredited Chest Pain Centers (CPCs) provide rapid and accurate diagnosis, risk assessment, and appropriate treatment for patients with acute chest pain [28]. In January 2016, the Chinese Cardiovascular Association launched the National Chest Pain Centers Program (NCPCP) to improve early diagnosis and standardized treatment of ACS and improve patients’ clinical outcomes (Supplemental Figure 1). The NCPCP is the first nationwide, comprehensive, hospital-based, continuous quality improvement (QI) program. CPC accreditation has five dimensions: (1) facility conditions, (2) diagnosis and treatment process, (3) integration of prehospital and hospital care, (4) training and education, and (5) capacity for continuous quality improvement. The NCPCP’s detailed design and interventions have been published previously [29]. In China, 1927 hospitals in 31 provinces have a certified CPC as of May 2021, and over 5000 hospitals have joined the NCPCP. However, the CPC distribution and the implementation process and intervention outcomes vary considerably across regions (Fig. 1).

Understanding the barriers and enablers in implementing a quality improvement program is critical for optimizing intervention strategies, extending the effect of implementation, and promoting intervention findings in other settings [30]. In this study, the Consolidated Framework for Implementation Research (CFIR) was used to investigate barriers and facilitators in NCPCP implementation in China. The CFIR is a determinant meta-theoretical framework that includes all constructs that interact across the implementation process and can be used to open the “black box” of implementation [31, 32]. The CFIR comprises 39 constructs organized into five major domains: characteristics of the organization implementing the intervention, factors external to the organization, characteristics of the intervention, characteristics of the individuals involved in implementation, and process of implementation (Supplemental Table 1) [31]. The CFIR was used throughout our research, from study design to data collection, analysis, and interpretation. With implementation and scaling up of the NCPCP in China, we aimed to (1) investigate barriers and enablers in NCPCP implementation and (2) provide examples and ideas for how to overcome these barriers.

## Methods

### Study design and population

In this study, we used multistage cluster sampling to select the CPCs in China. First, staff at the national headquarters of the Chest Pain Centers randomly chose six cities, Suzhou, Wuhan, Changsha, Hefei, Chongqing, and Shenzhen, to represent cities nationally according to differences of economic development and health resources as well as the degree of CPC development. Second, 20 hospitals with accredited CPCs in each city were randomly selected. All hospitals with CPCs in Hefei and Changsha were chosen because these cities had fewer than 20 such hospitals. Eventually, 90 CPCs with nationally representative of CPCs in China were chosen in our study.

From the 90 CPCs, each CPC director, who has a key decision-making role, and CPC coordinator, who has a transactional-working role, were invited to participate in semi-structured key informant interviews to investigate potential barriers, enablers, and ideas to overcome barriers in NCPCP implementation. CPC directors and coordinators were chosen because they were involved in the entire process of NCPCP implementation and were most likely to have in-depth knowledge of barriers and enablers in implementation and implementation effectiveness. The key informant interviewees from the nationally representative CPCs in China guaranteed our study captured all relevant themes and data about the barriers and enablers to NCPCP implementation. The Ethics Committee of Peking University Health Science Center approved this study (IRB00001052-21020). Written informed consent from respondents was obtained before they participated in the interviews, which were conducted between July and August 2021.

### Data collection

The interviews were semi-structured, with most consisting of an in-depth individual interview and the remainder consisting of a focus group discussion. All interviews were conducted by trained qualitative researchers (YJ, SZ, JM, XD, and NL; of whom, three were women) and were recorded by note-takers. The interview guide was developed based on the CFIR, and the guide’s final version was developed after two rounds of experts’ consultation consisting of cardiologists and health care researchers. The main content of the face-to-face interviews varied. Interviews with the director of each CPC focused on the external context of the program, internal setting, and intervention characteristics; interviews with the coordinator emphasized characteristics of individuals and the implementation process. The average length of each interview was approximately 50 min. Interviews were digitally recorded and conducted in the interviewees’ native Chinese dialect. The survey was administered in 90 hospitals with CPCs (Supplement Table 2), with 87 CPC directors and 78 CPC coordinators participating in the key informant interviews, and the response rate of the key informant interviews was 91.67%. The number of participants was sufficient for data saturation [33]; data saturation was reached because there was no new information obtained by the end of the interviews.

### Data management and analysis

All video recordings were transcribed verbatim and translated into English for analysis. NVivo 12.0 (QSR International, Melbourne, Australia) was used for theme coding. A well-trained author (SZ) analyzed the transcripts to get a sense of the data before coding, then used the content analysis method to code the data using the CFIR’s 39 constructs as the coding framework. To ensure interrater agreement, a second coder (YJ) independently coded 10% of the transcripts. The coders met regularly to resolve problems or disagreements and reached a consensus on coding. The data coded into each CFIR domain were then reviewed to generate the common assertions regarding barriers and enablers in NCPCP implementation. The Consolidated Criteria for Reporting Qualitative Studies (CQREQ) checklist was used for qualitative analysis by a research team to assess the study design, analysis, and findings [34]. The research team was from the Department of Global Health, Peking University School of Public Health.

## Results

### Characteristics of participants

One hundred sixty-five participants completed the interviews, among whom 110 were men; 78 were CPC directors. The average tenure among CPC directors was 21 years (range 15–26 years), whereas CPC coordinators had an intermediate term of 11 years (range 7–16 years). Over half of CPC coordinators had an attending title whereas nearly all CPC directors (81/87) had a chief title. Approximately 80% of CPC directors held master’s or doctoral degrees; the proportion was similar for CPC coordinators (Table 1).

**Table 1:** Demographic information of participants

Participants’ characteristics	CPC directors	CPC coordinators
Working year^a^	21 (15–26)	11 (7–16)
Gender^b^		
Male	68 (78.16)	42 (53.85)
Female	19 (21.84)	36 (46.15)
Title^b^		
Chief	81 (93.10)	32 (41.03)
Attending	6 (6.90)	43 (55.13)
Junior	0 (0.00)	3 (3.85)
Education^b^		
Doctoral	20 (22.99)	10 (12.82)
Master	52 (59.77)	44 (56.41)
Bachelor	15 (17.24)	24 (30.77)

*CPC* chest pain center

^a^ Median (IQR)

^b^ *n* (%)

### Barriers and enablers

**Fig. 2:**
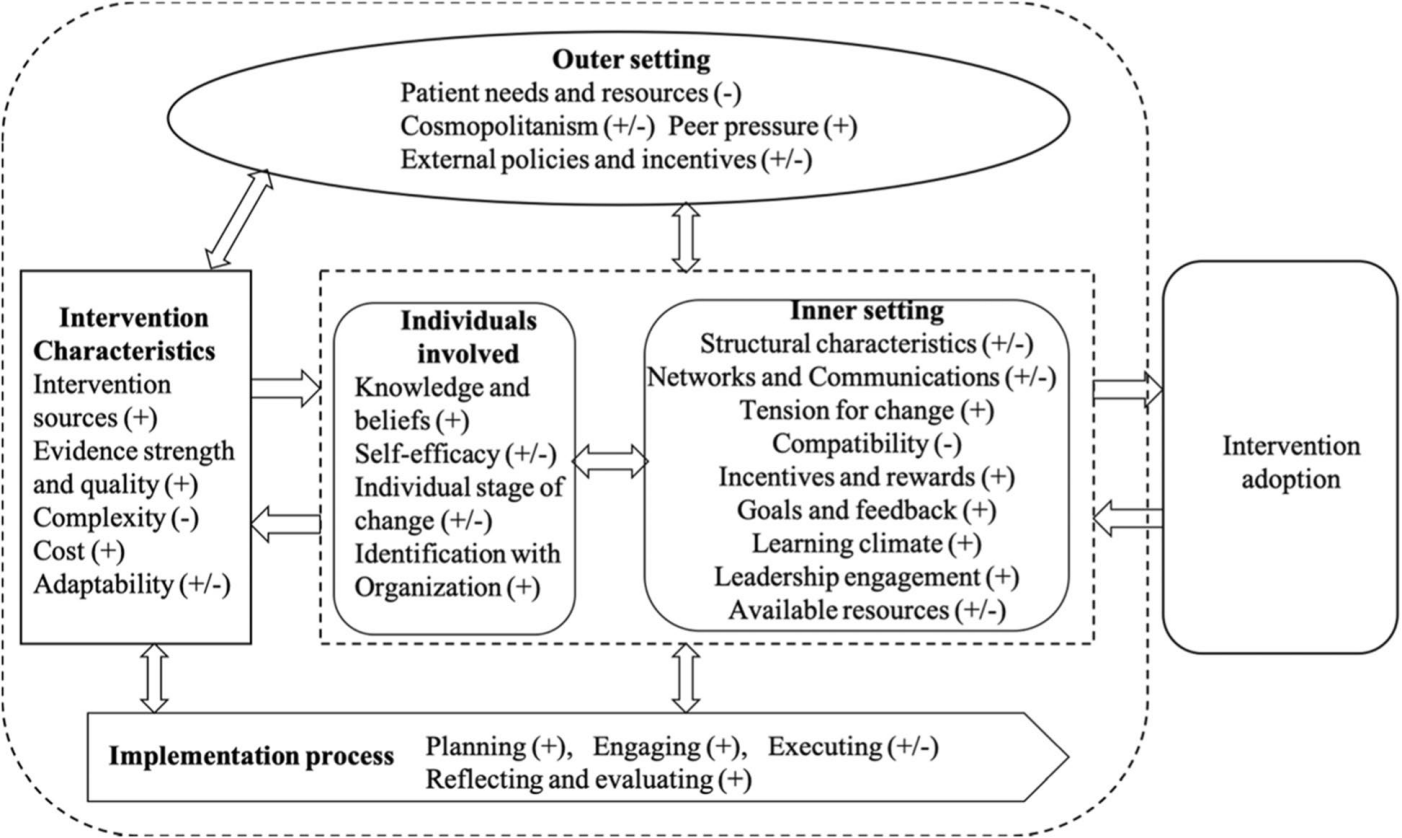
CFIR constructs that emerged in this study and their influence on implementation of NCPCP. *Note*: +, enablers; −, barriers. CFIR, Consolidated Framework for Implementation Research; NCPCP, National Chest Pain Centers Program

As Fig. 2 shows, we identified that 26 of the 39 CFIR constructs facilitated or impeded NCPCP implementation, covering 5 CFIR domains. Of these constructs, 3 were barriers, 14 were enablers, and 9 were both barriers and enablers. The five domains of the CFIR interacted with each other, providing a practical guide to systematically explore barriers and enablers in NCPCP implementation (Fig. 2).

### Characteristics of the intervention

#### Intervention sources

The intervention source indicated that the NCPCP was developed owing to both internal and external factors. From the perspective of hospitals, CPC accreditation fostered development of the cardiology discipline and multidisciplinary integration; this also reduced within-hospital medical disputes, improved the visibility and impact of the field, and attracted more patients. From the perspective of patients, through prehospital emergency and in-hospital resource integration, the treatment process for patients with chest pain was optimized, treatment time for those with ACS was shortened, and mortality and complications in patients with ACS were reduced. Government policies drove the external factors. Public hospitals were required to establish CPCs by the National Health Commission to the Provincial and Municipal Health Commission. For example, the Wuhan Health Commission required all tertiary hospitals to obtain CPC accreditation in 2017.

Three main reasons for participating the NCPC. First, the whole country, including the National Health Commission and the Provincial Health Commission, now administratively requires you to create CPCs. Second is the development of our disciplines. Third is from the people’s point of view, CPCs emphasize early treatment, and various departments provide a green channel to facilitate patient consultation, thus ultimately benefiting the public.

-Cardiology Chief, Chest Pain Center Director

#### Evidence strength and quality

The evidence regarding the strength and quality of intervention has been a key determinant in implementation. The NCPCP has indeed improved the care quality for patients with ACS, with a substantial reduction in in-hospital mortality and a significant decrease in the incidence of complications, such as heart failure and arrhythmias. Through CPC establishment, hospitals have improved the diagnosis and treatment levels for ACS, thus enhancing the reputation and influence of these hospitals. Additionally, the CPC establishment lays a solid foundation for the accreditation of stroke centers, atrial fibrillation centers, and trauma centers in hospitals and provides a model from which to learn. Furthermore, the CPC establishment is important in raising the level of emergency care, awareness, and capability of the entire hospital.

#### Complexity

Complexity is crucial for influencing intervention implementation, and CPC directors and coordinators expressed concerns about the program’s complexity. Complexity represented the subjective feelings of participants about the requirements of NCPCP. Informants stated that the accreditation criteria and procedures are complex, and the preparation of materials, including developing flowcharts, is time-consuming, which is not conducive to establishing CPCs in primary hospitals. According to interviewees, variable entries such as time points are too demanding and cumbersome in the operation process, particularly the collection and reporting of case data for patients with chest pain, and these form-completion tasks increase the burden for medical staff. ACS discharge follow-ups and other requirements are also reportedly complex, and data availability is poor, resulting in less motivation among medical staff and fewer forms completed.

For those of us who work at the bottom, data reporting is usually a headache. I'm sure if you ask anyone, they'll tell you the same thing. The data issue, in my opinion, is the most difficult for us. -Cardiologist, Chest Pain Center Coordinator

It implies that the more you complete, the more work you'll have to add to the local practitioner's schedule. We're all working for free, and these things don't immediately benefit us. Of course, filling out forms helps us be better at our job, but they're too time-consuming to complete.

-Cardiology Chief, Chest Pain Center Director

Interviewees stated that the accreditation criteria needs to be further simplified for different hospitals, especially for primary hospitals, and that capturing key indicators is sufficient.

#### Cost

The construction cost of a CPC includes purchasing equipment such as electrocardiography (ECG) equipment, troponin testers, and interventional equipment. Additionally, there are direct expenses such as salaries for the CPC’s data collection and form completion staff, and investment of time and energy by the cardiology department’s medical staff. For the department, this involves more indirect investment, such as staff time. CPC directors believed that CPC construction was cost-effective and worth promoting because of the positive social and economic benefits it achieved.

We didn't invest much money on this, only the time and effort that everyone put in throughout that period.

-Cardiology Chief, Chest Pain Center Director

#### Adaptability

To date, two versions of the CPC certification standard exist: the standard version for hospitals with percutaneous coronary intervention (PCI) capability and the basic version for hospitals without PCI capability. However, standardization of the CPC assessment standards has introduced new issues, such as the requirement regarding the bypass emergency ratio, which may lead to medical risks of misdiagnosis, and the requirement regarding in-hospital mortality, which may cause some hospitals to reject patients with severe conditions. Informants agreed that the standards for CPCs should be handled flexibly, following the actual situation of each hospital.

As with the earlier-mentioned issue of in-hospital mortality, the rate of in-hospital mortality sometimes exceeds the accreditation standards because the baseline is low in some hospitals. I believe there should be some flexibility for certain indicators to be combined according to the specific circumstances of each hospital.

–Cardiology Chief, Chest Pain Center Director

### External settings

#### Patient needs and resources

ACS patient-level factors are substantial contributors to delays in care. Understanding patients’ needs and influencing factors effectively reduces patient delays and facilitates NCPCP implementation. Patient delays are primarily reflected in the time between onset of chest pain to first medical contact, and delays obtaining pre-surgery informed consent. CPC directors and coordinators emphasized that multilevel strategies should be used to reduce patient delays. Media outlets should increase favorable and active coverage of stents and other medical devices for patients with ACS to dispel common misconceptions. Expert resources should be fully exploited to reach the grassroots level so as to promote awareness of the importance of timely consultation. Finally, to improve patient trust, informed patient consent should be optimized at the hospital level.

The biggest obstacle to implementation is still in the prehospital stage. Some patients have problems with awareness about chest pain; for example, you know that chest pain is an urgent situation, but the patient does not at all.

-Cardiology Chief, Chest Pain Center Director

#### Cosmopolitanism

A crucial factor affecting NCPCP implementation and effectiveness is the proximity of hospitals and lower-level medical facilities to the prehospital emergency system. With diverse emergency system models and cooperation mechanisms between hospitals and emergency systems, the degree of linkage between prehospital emergency and in-hospital treatment are critical determinants impacting the clinical outcome of patients with ACS. For lower-level medical institutions, timely diagnosis and rapid referral are keys to effectively shortening treatment delays. Establishing an efficient out-of-hospital cooperation mechanism is a dilemma in the process of CPC accreditation. Interviewees stated that the answer to this problem requires a top-level design from the government.

I'd want to make it a one-stop model for prehospital and in-hospital emergencies. But why does prehospital emergency systems only send patients to some hospitals and not others? Some hospitals may have a good connection with 120; thus, patients are referred to these hospitals.

-Cardiology Chief, Chest Pain Center Director

We can only control ourselves, from the entrance to the hospital gate; we can only shorten in-hospital delays. As for how to build up the network in other hospitals, this may still need to be improved by the government.

-Cardiology Chief, Chest Pain Center Director

#### External policies and incentives

Government policies and financial support are crucial to the successful CPC establishment. Government support for CPC accreditation varies, and it frequently remains at the stage of issuing documentation, with no further evaluation, which has a limited promotional effect. Inversely, some district or county health commissions place a high value on CPC construction, making this a priority for the entire district/county and providing financial and policy support to improve the level of emergency care.

The attention of some Municipal Health Commissions given to CPCs is insufficient, and they merely stay in the stage of document issuing. The Health Commission lacks the sense of urgency required to implement CPC accreditation.

-Cardiology Chief, Chest Pain Center Director

CPCs directors highlighted the need for further strengthening of government support in the routine operation of CPCs, particularly health education for community members; the interface between hospitals and emergency systems; and the construction of chest pain emergency networks.

#### Peer pressure

Peer pressure promotes NCPCP implementation to some extent. Hospitals with relatively better comprehensive strength have been prompted to join the NCPCP with the CPC accreditation in hospitals that have weaker technical capabilities. Simultaneously, hospitals or cardiology departments have consolidated their academic status and disciplinary reputation on a national or local level through CPC accreditation. As neighboring hospitals continue to obtain CPC accreditation, non-accredited hospitals are faced with the dilemma of diminished attractiveness and patient flow, further encouraging them to join the NCPCP.

Our hospital, whether in terms of academics or reputation, definitely wants to integrate into the collective and build chest pain centers together.

-Cardiologist, Chest Pain Center Coordinator

### Internal settings

#### Structural characteristics

Respondents reported that the organization’s structure and size plays an essential role in NCPCP implementation. Interviewees revealed that larger hospitals attach less importance to CPCs than smaller hospitals, especially larger general hospitals where patients with chest pain account for only 5% of all patients in the emergency department, making the initial CPC establishment relatively risky. The problem of mobility in the emergency department and other related departments can hamper NCPCP implementation. CPC directors suggested that staff mobility adversely affects several aspects, such as identifying and diagnosing atypical chest pain, and results in misreporting and omitting emergency data. However, this also reflects a lack of personnel training at CPCs in these hospitals.

What is the reason for the lack of training? It is because there is a lot of staff turnover in hospitals, with many recruits, including nursing staff, doctors, and housekeeping nurses. Hospital training for new staff in CPCs is insufficient.

-Cardiology Chief, Chest Pain Center Director

#### Networks and communications

One concept of the CPCs is to integrate hospital resources to provide timely treatment for patients with acute chest pain. CPC accreditation is a hospital-wide initiative, which requires the support and cooperation of related departments. During the interviews, we discovered that the emergency departments of most hospitals have shown some resistance to CPC construction, believing that a CPC is a matter for the cardiology department but that adds an extra workload to the emergency department. This resistance has had a significant impact on the process of promoting CPC accreditation.

Many departments may first believe that this is an issue of our cardiology department, and it is problematic for that department to initiate things like this. However, because of various forms of publicity, people are increasingly aware of the chest pain center, and that awareness has become more and more widespread.

-Cardiologist, Chest Pain Center Coordinator

Interviewees believed that the above problems could be solved in three ways: (1) improving communication with the emergency department so that emergency department staff understand the importance of CPC accreditation and its importance to the emergency department; (2) establishing a reward and punishment system at hospital level, and including routine CPC tasks in performance evaluation; (3) allowing the emergency department to fully participate in CPC accreditation and to reap the benefits, meaning that all patients with outpatient chest pain should go to the emergency department and start the process of chest pain evaluation in that department to increase its patient volume.

#### Implementation climate

From an endogenous perspective, hospitals that urgently need to improve the care quality for patients with ACS and reduce the occurrence of doctor–patient disputes by establishing a CPC are driven to join the NCPCP, and such hospitals have had a higher degree of fidelity to NCPCP interventions (tension for change). Some hospitals have provided incentives for staff in charge of data reporting. For example, the hospital provides approximately 100 Yuan to medical staff in the emergency department, cardiology department, and catheterization department for completing a medical record form for high-risk patients with chest pain (organizational incentives and rewards). CPC coordinators review the completed data and penalize medical staff for any errors or omissions (goals and feedback). Some hospitals have established a performance appraisal system based on the CPC evaluation index for medical staff who perform emergency PCI procedures and the staff of radiology departments; rewards or penalties are determined based on whether the standards are met (organizational incentives and rewards, goals and feedback). The implementation of these measures facilitates NCPCP implementation and ensures the fidelity to and effectiveness of the intervention. By joining the NCPCP, young doctors have considerably enhanced their diagnosis and surgical abilities and gained professional pleasure and pride through continuous learning (learning climate).

CPC coordinators, particularly doctors in tertiary institutions, handle numerous specific activities in regular CPC operations, including data review and holding quality analysis meetings and case discussion, which have had a substantial impact on their everyday medical, research, and teaching tasks (compatibility). In response to these issues, CPC directors and coordinators highlighted the importance of enhancing the level of information technology to free up medical staff. Simultaneously, further simplifying the criteria for CPC-related meetings and documents is important to reduce the workload at the source.

#### Readiness for implementation

Implementation of the NCPCP has received strong support from hospitals, including leadership engagement from hospital level to department level. Directors of cardiology and emergency departments in some hospitals have been removed from their positions after failing CPC accreditation (leadership engagement). To successfully pass accreditation, hospitals have provided funding, equipment, and personnel to cardiology and emergency departments. However, informants stated that after passing CPC accreditation, hospital support in routine CPCs, including incentive funding and personnel training, begins to decrease gradually (available resources).

In preparation for accreditation, there was clear support at a hospital-wide level, and our vice president attended almost every meeting related to CPC accreditation.

-Cardiology Chief, Chest Pain Center Director

### Characteristics of involved individuals

#### Knowledge and beliefs about the intervention

The recognition by medical staff of CPC effectiveness is an influential factor in promoting NCPCP implementation. Respondents perceived two effects of CPC establishment on patient treatment behavior among medical staff: first, more standardized treatment behavior, which effectively reduces the occurrence of missed diagnoses; second, more timely treatment, which effectively reduces the occurrence of cardiovascular-related complications in patients. Improved patient outcomes provide more intrinsic professional satisfaction to medical staff, motivating them to follow the NCPCP requirements in clinical practice.

I mean that the staff member can see his/her sense of achievement in doing something. They did save the patient, and this patient made an adequate recovery. The most important thing is that staff have a sense of accomplishment and satisfaction.

-Cardiologist, Chest Pain Center Coordinator

#### Self-efficacy

The NCPCP is a continuous quality improvement program that revolves around CPC certification. Hospitals have made concerted efforts in preparation for CPC accreditation, and key personnel, including CPC coordinators, have felt confident in successful accreditation. Additionally, the problems revealed during daily operations, including a lack of patient awareness, interfacing with the emergency system, and support for continuous operations, have required the support and participation of the government, community, and society.

#### Individual stages of change

Different departments and medical staff within the same department had different perspectives on the NCPCP. NCPCP implementation had an impact on patient treatment behavior among staff, which was the most difficult to change. However, as the NCPCP progressed, patients and hospitals benefited from it, identification with the NCPCP gradually strengthened among medical staff, and the interventions slowly became part of their routine. To promote change in treatment behaviors among medical staff, some hospitals have hired dedicated data reporters, which has further lowered the burden on medical staff and improved their recognition of the NCPCP.

The most significant barrier is that various people have varying levels of understanding of CPCs, and people continue to have different levels of engagement. However, we have become accustomed to it after doing it, and it has become a permanent working pattern.

-Cardiologist, Chest Pain Center Coordinator

#### Individual identification with the organization

Identification among medical staff with the hospital influences the level of commitment to the NCPCP. Some interviewees stated that the NCPCP has brought considerable social and economic benefit to the hospital. Medical staff have fully supported their hospital's decision to join the NCPCP and treat joining NCPCP as a personal responsibility.

### Implementation process

#### Planning

A comprehensive and detailed implementation plan is a facilitator of NCPCP implementation. CPC directors indicated that hospitals that were interested in joining the NCPCP should focus on learning experiences and lessons from hospitals that have previously passed CPC accreditation. Hospitals should implement policies that facilitate the construction of CPCs and establish institutional assurances for CPC accreditation. The criteria for CPC accreditation should be clarified, and materials such as flow charts and clock unification for time points should be prepared following the standards. Training plays a vital role in NCPCP implementation, which includes training organized by national headquarters of the CPCs and provincial CPCs, training conducted at hospital level for relevant departments and all hospital staff, and training for community residents related to ACS. Finally, hospitals should concentrate on the prehospital setting and develop an effective interface mechanism with the prehospital emergency system and network hospitals.

#### Engaging

Building an executive team is critical to NCPCP implementation. The hospital president, who has overall responsibility; the chiefs of cardiology and emergency medicine, who are in charge of operations; and the chief of the medical administration office, who is in charge of coordination should be part of the CPC organizational architecture. However, leadership participation at the hospital level is insufficient, and supervision by government is also needed for CPCs to operate smoothly. CPC directors stated that the establishment of CPCs required the director of the cardiology department to have solid coordination and communication skills.

When we establish a CPC, the hospital director should be involved since more departments and employees are involved, and it is no longer a question of one person or a specific department. Second, we should rely on the government to closely monitor operational issues so that CPCs can function more efficiently.

-Cardiology Chief, Chest Pain Center Director

#### Executing

The most challenging aspect of NCPCP implementation was reported by respondents to be ensuring intervention fidelity in hospitals once a CPC had been accredited. Interviewees highlighted that some hospital leaders felt they had achieved their goals and support for CPC routine operations diminished after accreditation. Furthermore, a level of inertia existed among medical staff, including omissions when completing data forms for patients with chest pain. Reporting of chest pain patient data was still manual in most hospitals, with a low information technology penetration rate. The reasons for this include the high price of information systems and ineffective connection among the CPC data reporting system, hospital information system, and national CPC data reporting platform. Additionally, interviewees perceived inadequate implementation of CPC training, including training for new staff and for community-dwelling patients with chest pain. CPC directors emphasized the importance of the attitude and responsibility of the person in charge, and that CPC directors should promptly identify and address operational problems to achieve continuous quality improvement.

In our hospital, some leaders are still caught in not caring after obtaining certification. Some CPC signs are broken, and we've called to repair them. However, when calling from our level, the results are not very good. Some signs, such as a damaged lightbox, should be checked regularly.

-Cardiology Chief, Chest Pain Center Director

#### Reflecting and evaluating

Implementation of the NCPCP interventions is related to sustainability of the effect of CPC establishment. CPC directors proposed that self-inspection and external inspection be used to supervise the routine operation of CPCs. For hospital self-inspection, CPC directors felt that attention was needed regarding quality control and timely correction of deviations. The national headquarters of the CPCs should supervise the NCPCP implementation interventions in hospitals through unannounced visits and regular inspections. Governments can also act as a strong promoter of external assessment by organizing administratively binding CPC quality control centers.

## Discussion

Using the CFIR, in this qualitative study, we explored the perceptions of CPCs directors and coordinators regarding barriers and enablers in implementing an ongoing national ACS quality improvement initiatives in China. Identified barriers to the NCPCP implementation were the complexity of the intervention, low flexibility of requirements, a lack of recognition of chest pain in patients with ACS, relatively low government support for CPC implementation, staff mobility in the emergency department and other related departments, resistance from associated departments, overwhelming tasks for CPC coordinators, lack of available resources for routine CPC operations, and fidelity and sustainability for intervention implementation. In contrast, enablers of intervention implementation were medical staff’s inner motivation for change, evidence on the strength and quality of the intervention, relatively low cost for cardiology departments, medical staff knowledge and beliefs regarding the intervention, stress from other hospitals, hospital incentives and rewards for the intervention, and involvement of hospital leaders. These barriers and enablers are in line with the findings of other related studies [35, 36]. To our knowledge, this study is the first process evaluation of an ongoing nationwide ACS quality improvement program in China. The findings will generate actionable knowledge to inform the design of impactful and sustainable quality improvement initiatives for ACS in China and other developing countries.

The implementation of NCPCP is a complex process by which the interventions were used in CPCs routinely [31]. The five domains of the CFIR framework provided the comprehensive factors associated with the implementation process of NCPCP. Previous studies pointed out that the implementation is a social process that interacts with the contextual factors both from the inner and outer settings [37, 38]. In our study, factors associated with the inner settings known as the characteristics of the CPCs joined the NCPCP, such as the communications and networks between different departments and the supportive implementation climate of hospitals, all influence implementation. Factors external to the CPCs, such as the policy from governments and pressure from other hospitals, promoted the implementation of NCPCP to some degree. Meanwhile, whether the interventions are adaptable to the work process of medical staff, the inner motivation of the medical staff for change, and the implementation process are all critical to the successful implementation of NCPCP.

Among hospital-based QI initiatives, the complexity of intervention is a major barrier to intervention implementation [39, 40]. The complexity of the NCPCP is reflected in the accreditation criteria and routine operational process. In preparing for accreditation, various written materials, flow charts, and patient case data preparation are involved; in the operating procedures, the complexity is reflected in data collection and reporting for patients with ACS. The data variables are very cumbersome, imposing a relatively heavy burden on medical staff when reporting these data and requiring a lot of time. In addition to daily clinical and scientific tasks, CPC coordinators take additional time to review the data and organize regular CPC meetings, which has a considerable impact on their daily work and affects their enthusiasm for CPC accreditation [41].

In patients with ACS, patient delays are highly associated with in-hospital mortality and comorbidities [42]. Identifying the best approach to reducing patient delays is a key element in facilitating NCPCP implementation. The prehospital focus in the NCPCP has been inadequate, with medical staff playing a very limited role in community training. There is a lack of adequate policy and resource support during NCPCP implementation, and raising patient awareness about chest pain emergencies is a social project that requires the collaborative efforts of government, communities, and medical staff [43]. In terms of patient informed consent, Chinese patients tend to have “family autonomy” [44], where the family plays an important role in medical decisions [45]. In Western countries, autonomy is purely individualistic, and patients’ wishes and preferences take a leading role in deciding treatment [46]. In the unique culture of China, doctors consult with the entire family to obtain consent for patient treatment [47]. Therefore, NCPCP-participating hospitals must enhance training, improve medical staff communication skills, and shorten the time to obtain patient informed consent [48].

Government support is particularly important in QI initiatives [49]. In NCPCP implementation, government support is reflected in both funding and coordination; financial support allows CPCs to have an adequate foundation of equipment and reserve personnel, and coordinated support is crucial for establishing a collaborative chest pain treatment network. The effective operation and seamless integration of the prehospital emergency system and the in-hospital treatment system is the decisive factor in shortening system-related delays for patients with ACS [49, 50]. Government support is key to optimizing the prehospital and in-hospital emergency care systems. In this study, we found that NCPCP support from different regional governments varies greatly and is closely related to regional economic development and the importance placed on support by leaders. Narrowing the policy support gaps in different regions is an essential issue for the NCPCP implementation in the future.

Previous studies have pointed out that the more stable an organization, the more successful implementation will be [51, 52], and this was validated in our study. Department staff mobility inhibited NCPCP intervention from being effectively communicated to relevant individuals. Reducing medical staff mobility and improving the frequency and quality of CPC training is key to successful implementation. The effectiveness of communication between different departments is related to the degree of agreement on the value of the intervention [53]. It is critically important to build an effective communication mechanism between departments through institutional constraints (formal approach) and daily interpersonal communication (non-confirmation process) to work toward goals.

CPC accreditation is the first phase of the NCPCP, which is an ongoing QI initiative. As a result, continuous resource support is needed to ensure intervention fidelity and the sustainability of intervention outcomes [54]. We found that CPCs experienced varying degrees of laxity after accreditation. Thus, there is a need to ensure the sustainability of resource support and maintain fidelity to implementation via supervision of multiple parties [55].

There are several strengths and limitations of our study. First, among the strengths, we chose a nationally representative sample of CPCs from six cities and key informants of the selected CPCs involved in the whole process of NCPCP implementation to ensure the generalizability of our results to the entire country. Second, using the CFIR, we obtained a comprehensive understanding of barriers and enablers in the NCPCP implementation process to guide the refinement of ongoing QI initiatives and allow for cross-comparisons with other settings. Third, whereas many studies have investigated barriers and enablers in implementing QI initiatives, our study also provided examples of how to overcome barriers. In terms of limitations, we did not obtain the viewpoints of other key stakeholders, such as patients and government officials. Furthermore, this study was descriptive, which limited our ability to determine a causal relationship. Future studies should include the perspectives of additional stakeholders to explore how to address implementation barriers, and quantitative studies are needed to evaluate the effects of overcoming the identified barriers on patient outcomes.

## Conclusion

Using the CFIR, we identified barriers and enablers in NCPCP implementation in China and ideas of how to address the identified barriers. Implementation of the NCPCP has benefited patients with ACS, hospitals, and society. Yet, how to maintain fidelity to implementation of the intervention is critical to the sustainability of the NCPCP. Simplifying the intervention to adapt to routine tasks for medical staff and optimizing the operation mechanisms between the prehospital emergency system and in-hospital treatment system with the support of the government, as well as enhancing emergency awareness of patients with chest pain are critically important to NCPCP implementation. Clarifying and addressing these barriers is key to designing a sustainable QI program for acute cardiovascular diseases in China and similar contexts across developing countries worldwide.

## Supplementary Information

**Additional file 1: Supplemental Figure 1.** The Chest Pain Center Accreditation Workflow in the NCPCP.

**Additional file 2.** CFIR domains and constructs within each domain.

**Additional file 3: Supplemental Table 1**. List of hospitals in our study.

**Additional file 4.** Interview guides.

## Data Availability

Please contact the corresponding author for more information.

## Abbreviations

IHD: Ischemic heart disease
ACS: Acute coronary syndromes
QI: Quality improvement
NCPCP: National Chest Pain Center Program
CPC: Chest pain center
CFIR: Consolidated framework for implementation research
PCI: Percutaneous coronary intervention
ECG: Electrocardiography
