# Supplementary figures and images for: Barriers and enablers in the implementation of a quality improvement program for acute coronary syndromes in hospitals: a qualitative analysis using the consolidated framework for implementation research
Source: Implement Sci. 2022 Jun 1;17:36. doi: 10.1186/s13012-022-01207-6 (PMC9158188; doi:10.1186/s13012-022-01207-6)

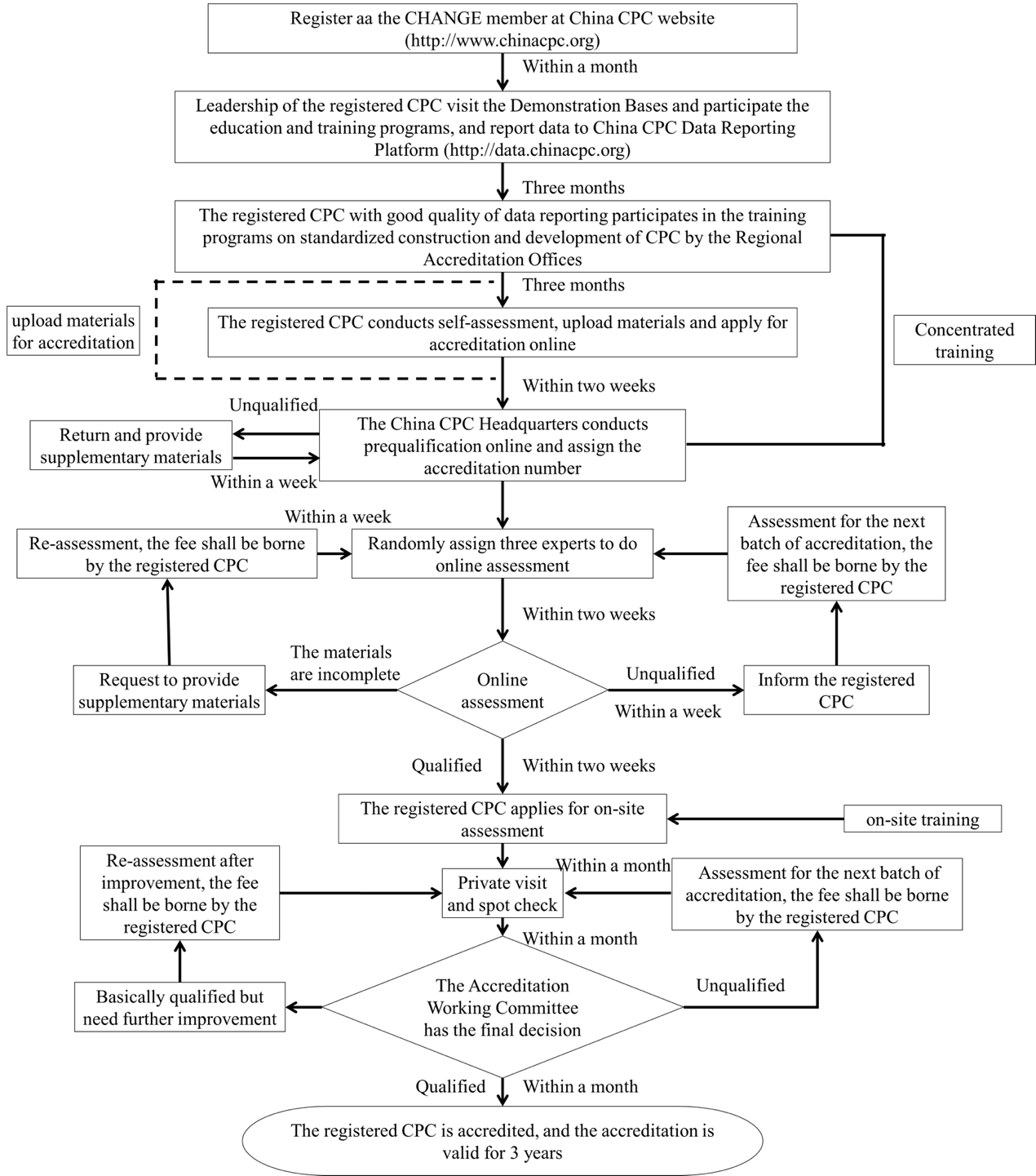


**Supplemental Fig 1.** The Chest Pain Center Accreditation Workflow in the NCPCP

Supplement: Supplementary file 1 — Additional file 1: Supplemental Figure 1. The Chest Pain Center Accreditation Workflow in the NCPCP. [file 13012_2022_1207_MOESM1_ESM.docx]
