# Supplementary material for: Barriers and enablers in the implementation of a quality improvement program for acute coronary syndromes in hospitals: a qualitative analysis using the consolidated framework for implementation research
Source: Implement Sci. 2022 Jun 1;17:36. doi: 10.1186/s13012-022-01207-6 (PMC9158188; doi:10.1186/s13012-022-01207-6)
Supplement: Supplementary file 2 — Additional file 2. CFIR domains and constructs within each domain. [file 13012_2022_1207_MOESM2_ESM.docx]

**CFIR domains and constructs within each domain**

| **Domain 1: Intervention –** characteristics of the intervention |
| --- |
| - Intervention source: Perception about whether intervention is externally or internally developed - Evidence Strength & Quality: Perception of the quality and validity of evidence supporting the belief that the intervention will have desired outcomes - Relative Advantage: Perception of the advantage of implementing the intervention versus an alternative solution - Adaptability: Degree to which an intervention can be tailored to meet the needs of an organization - Trialability: Ability to test the intervention on a small scale, and to reverse course if warranted - Complexity: Perceived difficulty of implementation - Design Quality & Packaging: Perceived excellence in how the intervention is bundled and presented - Cost: Cost of the intervention and costs associated with implementing the intervention |
| **Domain 2:** **Outer Setting –** factors external to the organization |
| - Patient Needs & Resources: Extent to which patient needs are accurately known and prioritized by the organization - Cosmopolitanism: Level of connectedness and networks with other organizations - Peer Pressure: Competitive pressure to implement an intervention - External Policy & Incentives: external strategies to spread interventions, including policy and regulations, mandates, recommendations and guidelines, etc. |
| **Domain 3: Inner Setting -** characteristics of the organization implementing the intervention |
| - Structural characteristics: Age, maturity, or size of the organization - Networks & Communication: Nature and quality of webs of social networks and the nature and quality of formal and informal communications within an organization - Culture: Norms, values, and basic assumptions of a given organization - Implementation climate: Relative priority of implementing the current intervention versus other competing priorities - Readiness for Implementation: Access to resources, knowledge, and information about the intervention |
| **Domain 4: Individuals -** characteristics of the individuals involved in implementation |
| - Knowledge and Beliefs about Intervention: Individual staff knowledge and attitude towards the intervention - Self-efficacy: An individual’s belief in their capabilities to execute the implementation - Individual State of Change: Phase an individual is in as he or she progresses toward skilled, enthusiastic, and sustained use of the intervention - Individual Identification with Organization: Individuals’ perception of the organization and their relationship and degree of commitment to the organization - Other Personal Attributes: Personal traits such as tolerance of ambiguity, intellectual ability, motivation, etc. |
| **Domain 5: Process –** processes of implementation |
| - Planning: Planning for the implementation - Engaging: Engaging individuals in implementation processes - Executing: Executing the implementation plan - Reflecting & Evaluating: Reflecting and evaluating the progress of implementation |
