# Supplementary material for: Barriers and enablers in the implementation of a quality improvement program for acute coronary syndromes in hospitals: a qualitative analysis using the consolidated framework for implementation research
Source: Implement Sci. 2022 Jun 1;17:36. doi: 10.1186/s13012-022-01207-6 (PMC9158188; doi:10.1186/s13012-022-01207-6)
Supplement: Supplementary file 3 — Additional file 3: Supplemental Table 1. List of hospitals in our study. [file 13012_2022_1207_MOESM3_ESM.docx]

**Supplemental Table 1 List of hospitals in our study**

| ID | Hospital | Province | City/County | Accreditation type | Accreditation date |
| --- | --- | --- | --- | --- | --- |
| 1 | Anhui Provincial Hospital | Anhui | Hefei | Comprehensive | 2017/4/20 |
| 2 | The Second Hospital of Anhui Medical University | Anhui | Hefei | Comprehensive | 2020/9/23 |
| 3 | The First Hospital of Anhui Medical University | Anhui | Hefei | Comprehensive | 2021/4/13 |
| 4 | The Second People's Hospital of Hefei | Anhui | Hefei | Comprehensive | 2019/10/31 |
| 5 | The First People's Hospital of Hefei | Anhui | Hefei | Comprehensive | 2018/11/2 |
| 6 | Binhu Hospital of Hefei | Anhui | Hefei | Comprehensive | 2021/4/13 |
| 7 | The Eighth Affiliated Hospital,Sun Yat-Sen University | Guangdong | Shenzhen | Comprehensive | 2014/12/12 |
| 8 | Central Hospital of Shenzhen Longhua District | Guangdong | Shenzhen | Comprehensive | 2017/9/2 |
| 9 | People's Hospital of Shenzhen Yantian District | Guangdong | Shenzhen | Comprehensive | 2019/4/11 |
| 10 | Shenzhen Traditional Chinese Medicine Hospital | Guangdong | Shenzhen | Comprehensive | 2021/5/17 |
| 11 | The Third Hospital of Shenzhen Longgang District | Guangdong | Shenzhen | Basic | 2019/12/12 |
| 12 | Peking University Shenzhen Hospital | Guangdong | Shenzhen | Comprehensive | 2019/4/11 |
| 13 | Central Hospital of Shenzhen Baoan District | Guangdong | Shenzhen | Basic | 2021/4/13 |
| 14 | Union Shenzhen Hospital (Nanshan Hospital) | Guangdong | Shenzhen | Comprehensive | 2017/12/14 |
| 15 | University of Chinese Academy of Sciences Shenzhen Hospital | Guangdong | Shenzhen | Comprehensive | 2021/4/13 |
| 16 | Shenzhen Sun Yat-Sen Cardiovascular Hospital | Guangdong | Shenzhen | Comprehensive | 2015/4/10 |
| 17 | People's Hospital of Shenzhen | Guangdong | Shenzhen | Comprehensive | 2015/7/23 |
| 18 | Shiyan People's Hospital, Baoan District, Shenzhen | Guangdong | Shenzhen | Basic | 2019/12/12 |
| 19 | Shenzhen Shekou People's Hospital | Guangdong | Shenzhen | Basic | 2018/8/6 |
| 20 | People's Hospital of Shenzhen Longgang District | Guangdong | Shenzhen | Comprehensive | 2016/11/4 |
| 21 | People's Hospital of Shenzhen Luohu District | Guangdong | Shenzhen | Basic | 2020/9/23 |
| 22 | Central Hospital of Shenzhen Longgang District | Guangdong | Shenzhen | Comprehensive | 2020/11/16 |
| 23 | People's Hospital of Shenzhen Baoan District | Guangdong | Shenzhen | Comprehensive | 2018/12/14 |
| 24 | The University of Hong Kong-Shenzhen Hospital | Guangdong | Shenzhen | Comprehensive | 2019/4/11 |
| 25 | Traditional Chinese Medicine Hospital Baoan, Shenzhen | Guangdong | Shenzhen | Basic | 2018/11/2 |
| 26 | BenQ Medical Center | Jiangsu | Suzhou | Basic | 2019/7/14 |
| 27 | The First People's Hospital of Kubnshan | Jiangsu | Suzhou | Comprehensive | 2016/7/24 |
| 28 | The First People's Hospital of Taicang | Jiangsu | Suzhou | Comprehensive | 2020/9/23 |
| 29 | Taicang Hospital of Traditional Chinese Medicine | Jiangsu | Suzhou | Basic | 2019/7/14 |
| 30 | Traditional Chinese Medicine Hospital of Kunshan | Jiangsu | Suzhou | Comprehensive | 2016/7/24 |
| 31 | The People's Hospital of SND | Jiangsu | Suzhou | Basic | 2018/11/2 |
| 32 | Huqiao Hospital of Kunshan | Jiangsu | Suzhou | Basic | 2017/11/3 |
| 33 | Kunshan NO.3 People's Hospital | Jiangsu | Suzhou | Basic | 2019/10/31 |
| 34 | Suzhou industrial park, Xinghai hospital | Jiangsu | Suzhou | Basic | 2017/12/14 |
| 35 | Suzhou Municipal Hospital | Jiangsu | Suzhou | Comprehensive | 2016/3/17 |
| 36 | Qiandeng Hospital of Kunshan | Jiangsu | Suzhou | Basic | 2018/12/14 |
| 37 | Suzhou industrial park, Xinghu hospital | Jiangsu | Suzhou | Basic | 2018/12/14 |
| 38 | Suzhou Kowloon Hospital, Shanghai Jiaotong University Medical School | Jiangsu | Suzhou | Comprehensive | 2014/12/12 |
| 39 | Kunshan NO.4 People's Hospital | Jiangsu | Suzhou | Basic | 2019/4/11 |
| 40 | The Second People's Hospital of Kunshan | Jiangsu | Suzhou | Basic | 2020/9/23 |
| 41 | The Second Affiliated of Soochow University | Jiangsu | Suzhou | Comprehensive | 2016/11/4 |
| 42 | Kunshan NO.6 Hospital | Jiangsu | Suzhou | Basic | 2018/12/14 |
| 43 | Suzhou Hospital Of Traditional Chinese Medicine | Jiangsu | Suzhou | Basic | 2021/4/13 |
| 44 | The Central Hospital of Wuhan | Hubei | Wuhan | Comprehensive | 2016/11/4 |
| 45 | Wuhan Third Hospital-Tongren Hospital of Wuhan University | Hubei | Wuhan | Comprehensive | 2017/7/22 |
| 46 | Fifth Hospital in Wuhan | Hubei | Wuhan | Comprehensive | 2017/4/20 |
| 47 | CR & WISCO General Hospital | Hubei | Wuhan | Comprehensive | 2017/4/6 |
| 48 | Puai Hospital of Wuhan | Hubei | Wuhan | Comprehensive | 2017/9/2 |
| 49 | Wuhan NO.1 Hospital\|Wuhan Hospital Of Chinese And Western Medicine | Hubei | Wuhan | Comprehensive | 2017/9/2 |
| 50 | Puren Hospital of Wuhan | Hubei | Wuhan | Comprehensive | 2017/4/6 |
| 51 | Red Cross Hospital of Wuhan | Hubei | Wuhan | Basic | 2018/8/6 |
| 52 | Union Hospital affiliated to Tongji Medical College of Huazhong University of Science and Technology | Hubei | Wuhan | Comprehensive | 2016/11/4 |
| 53 | Wuhan NO.6 Hospital | Hubei | Wuhan | Basic | 2019/12/12 |
| 54 | Wuhan Hankou Hospital | Hubei | Wuhan | Basic | 2020/9/23 |
| 55 | The Third People's Hospital of Hubei Province | Hubei | Wuhan | Comprehensive | 2018/12/14 |
| 56 | Wuhan Hanyang Hospital | Hubei | Wuhan | Basic | 2017/12/14 |
| 57 | Renmin Hospital of Wuhan University | Hubei | Wuhan | Comprehensive | 2017/7/22 |
| 58 | Wuhan ASIA Heart Hospital | Hubei | Wuhan | Comprehensive | 2014/12/12 |
| 59 | Tongji Hospital, Tongji Medical College, Huazhong University of Science & Technology | Hubei | Wuhan | Comprehensive | 2015/11/13 |
| 60 | General Hospital of The Yangtze River Shipping | Hubei | Wuhan | Basic | 2017/9/2 |
| 61 | Wuhan Hospital of Traditional Chinese Medicine | Hubei | Wuhan | Basic | 2019/12/12 |
| 62 | Wuhan Wuchang Hospital | Hubei | Wuhan | Basic | 2018/8/6 |
| 63 | Zhongnan Hospital of Wuhan University | Hubei | Wuhan | Comprehensive | 2017/9/2 |
| 64 | Renmin Hospital of Hunan | Hunan | Changsha | Comprehensive | 2017/4/6 |
| 65 | The Second People's Hospital of Changsha | Hunan | Changsha | Basic | 2019/7/14 |
| 66 | The People's Hospital of Liuyang | Hunan | Changsha | Comprehensive | 2019/7/14 |
| 67 | Changsha Center Hospital | Hunan | Changsha | Comprehensive | 2017/12/14 |
| 68 | Xingsha Hospital of Changsha | Hunan | Changsha | Basic | 2021/4/13 |
| 69 | The Second Xiangya Hospital of Center South Univerity | Hunan | Changsha | Comprehensive | 2018/12/14 |
| 70 | The First Hospital of Changsha | Hunan | Changsha | Comprehensive | 2020/9/23 |
| 71 | Traditional Chinese Medicine Hospital of Liuyang | Hunan | Changsha | Basic | 2019/10/31 |
| 72 | Hunan Aerospace Hospital | Hunan | Changsha | Basic | 2019/10/31 |
| 73 | The Fourth Hospital of Changsha | Hunan | Changsha | Comprehensive | 2021/4/13 |
| 74 | People's Hospital of Chongqing Banan District | Chongqing | Banan | Basic | 2017/11/3 |
| 75 | People's Hospital of Chongqing Qijiang District | Chongqing | Qijiang | Basic | 2020/11/16 |
| 76 | Chongqing Emergency Medical Center | Chongqing | Yuzhong | Comprehensive | 2018/3/22 |
| 77 | People's Hospital of Chongqing Wansheng Economic Development Zone | Chongqing | Qijiang | Basic | 2017/4/6 |
| 78 | Yongchuan Hospital of Chongqing Medical University | Chongqing | Yongchuan | Comprehensive | 2017/4/6 |
| 79 | The People's Hospital of Dazu, Chongqing | Chongqing | Dazu | Comprehensive | 2018/12/14 |
| 80 | Chongqing Fuling Central Hospital | Chongqing | Fuling | Comprehensive | 2020/9/23 |
| 81 | Chongqing Fengdu County People's Hospital | Chongqing | Fengdu | Basic | 2018/12/14 |
| 82 | The First Hospital of Chongqing Medical University | Chongqing | Yuzhong | Comprehensive | 2016/7/24 |
| 83 | Chongqing Fifth People's Hospital | Chongqing | Nanan | Comprehensive | 2018/12/14 |
| 84 | Chongqing Jiangjin District Central Hospital | Chongqing | Jiangjin | Comprehensive | 2021/4/13 |
| 85 | Chonggang General Hospital | Chongqing | Dadukou | Basic | 2018/11/2 |
| 86 | General Hospital of Chongqing Nantong Mining Co. | Chongqing | Qijiang | Basic | 2019/4/11 |
| 87 | People's Hospital of Chongqing Rongchang District | Chongqing | Rongchang | Comprehensive | 2020/9/23 |
| 88 | Chongqing Dianjiang County Hospital of Traditional Chinese Medicine | Chongqing | Dianjiang | Basic | 2021/4/13 |
| 89 | The Third Affiliated Hospital of the Third Military Medical University | Chongqing | Yuzhong | Comprehensive | 2017/7/22 |
| 90 | Chongqing Dianjiang County People's Hospital | Chongqing | Dianjiang | Basic | 2019/12/12 |
