# Supplementary material for: Barriers and enablers in the implementation of a quality improvement program for acute coronary syndromes in hospitals: a qualitative analysis using the consolidated framework for implementation research
Source: Implement Sci. 2022 Jun 1;17:36. doi: 10.1186/s13012-022-01207-6 (PMC9158188; doi:10.1186/s13012-022-01207-6)
Supplement: Supplementary file 4 — Additional file 4. Interview guides. [file 13012_2022_1207_MOESM4_ESM.docx]

**Interview Guides for the NCPCP Process Evaluation**

***CPCs Coordinators Interview Guide***

1. Please give a brief description of your background, including education, title, specialty, and years of experience.

2. When was your hospital accredited as a chest pain center, when did you start reporting data, and what did the hospital do when the data started to be reported?

3. What activities are you personally involved in for the Chest Pain Center Quality Interventions?

4. What is the specific implementation status of the chest pain center quality interventions in our hospital? (Chest Pain Center accreditation, data reporting, quality review and feedback, education and training activities for health care professionals)

5. How do you view the Chest Pain Center Quality Improvement Program? Can improve the quality of ACS patients? What changes can it bring to you personally and to your unit? (income, career gain, doctor-patient relationship, reputation of the hospital)

6. Are you familiar with the guidelines for treating ACS patients in your daily practice? Do you think the guidelines for the treatment of ACS patients can improve the quality of patient care? How well do you personally implement them?

7. How do you think the implementation of Chest Pain Center Certification has affected your treatment behavior? Please give specific examples.

8. How do you think the quality of care for patients with acute coronary syndrome has changed since the implementation of the Chest Pain Center accreditation? What are the main reasons for the changes? (patient's perspective, physician's perspective)

9. What problems exist in the daily data reporting and management process? How do you overcome these problems? (Reporting methods, frequency, etc.)

10. Do you think the implementation effect of the chest pain center certification has met your expectation and what factors have facilitated/hindered the implementation of the quality improvement plan and the implementation of the plan? (internal environment, external environment, complexity of measures, recognition of personnel, fidelity of implementation, etc.)

11. What do you think are the problems with the chest pain center certification itself (is it complex? Does it affect the daily work, training work, etc.)? What are the suggestions for future improvement?

12. What do you think is the cost effectiveness of chest pain center certification? Are the effects of accreditation sustainable? Why?

***CPCs Directors Interview Guide***

1. Please briefly introduce your background, including your position, title, specialty, years of experience, etc.

2. When did your hospital become accredited? What were the reasons for deciding to join the Chest Pain Center Accreditation in (Policy? Intrinsic need?)

3. what did the hospital and the department do to prepare and work for the accreditation of the chest pain center?

4. Did you have sufficient resources and policy support (including support from the health care commission and hospital level) in the process of building the chest pain center?

5. In the process of the construction of the chest pain center, how do the staff of the relevant departments in the hospital view the construction of the chest pain center, and what is the attitude and motivation of the staff? (agree, need, cooperate with hospital action)

6. In the process of the construction of the chest pain center, what is the specific implementation of the requirements in this hospital? Are they implemented according to the regulations and plans? (Chest pain center accreditation, data reporting, quality review and feedback, education and training activities for medical and nursing staff) What measures does your hospital take to promote the implementation and enforcement of chest pain center accreditation? (Work model adjustments, incentives, etc.)

7. Who is responsible for data reporting? How is it implemented? What problems exist in the process of data reporting and management? How has your facility overcome these problems?

8. What internal policy and procedural changes have been made at your institution to reduce pre-hospital delays? What internal policy and procedural changes have been made at your facility to reduce in-hospital delays? (e.g., EMS-Emergency Department-Cardiology linkage)

9. How do you feel about Chest Pain Center accreditation? What changes can it bring to you personally and to your unit? (income, sense of professional gain, doctor-patient relationship, reputation of the hospital)

10. How do you think the quality of care for patients with acute coronary syndrome has changed since the implementation of chest pain center certification? What are the main reasons for the changes?

11. What do you think is the cost effectiveness of chest pain center certification? Are the effects of accreditation sustainable? Why?

12. Do you think the implementation of the chest pain center certification has been as effective as you expected and what factors have facilitated/hindered the implementation of the quality improvement program and the implementation of the program? (internal environment, external environment, complexity of measures, buy-in of personnel, fidelity of implementation, etc.)

13. Has accreditation of chest pain centers become an institutionalized design and arrangement for hospitals? What are the experiences and lessons learned that can be shared with other chest pain centers?

14. What problems have hospitals encountered in the process of building chest pain centers? What are the suggestions for improvement for the future development of chest pain centers?
